# FANCJ protein is important for the stability of FANCD2/FANCI proteins and protects them from proteasome and caspase-3 dependent degradation

**DOI:** 10.18632/oncotarget.5006

**Published:** 2015-08-21

**Authors:** David W. Clark, Kaushlendra Tripathi, Josephine C. Dorsman, Komaraiah Palle

**Affiliations:** ^1^ Department of Oncologic Sciences, Mitchell Cancer Institute, University of South Alabama, Mobile, Alabama, USA; ^2^ Department of Clinical Genetics, Section Oncogenetics, VU University Medical Center, Amsterdam, The Netherlands

**Keywords:** Fanconi anemia, FANCJ, FANCD2, caspase-3, proteasome

## Abstract

Fanconi anemia (FA) is a rare genome instability syndrome with progressive bone marrow failure and cancer susceptibility. *FANCJ* is one of 17 genes mutated in FA-patients, comprises a DNA helicase that is vital for properly maintaining genomic stability and is known to function in the FA-BRCA DNA repair pathway. While exact role(s) of FANCJ in this repair process is yet to be determined, it is known to interact with primary effector FANCD2. However, FANCJ is not required for FANCD2 activation but is important for its ability to fully respond to DNA damage. In this report, we determined that transient depletion of FANCJ adversely affects stability of FANCD2 and its co-regulator FANCI in multiple cell lines. Loss of FANCJ does not significantly alter cell cycle progression or FANCD2 transcription. However, in the absence of FANCJ, the majority of FANCD2 is degraded by both the proteasome and Caspase-3 dependent mechanism. FANCJ is capable of complexing with and stabilizing FANCD2 even in the absence of a functional helicase domain. Furthermore, our data demonstrate that FANCJ is important for FANCD2 stability and proper activation of DNA damage responses to replication blocks induced by hydroxyurea.

## INTRODUCTION

Fanconi Anemia (FA) is a rare, inherited genetic instability disorder that is characterized by developmental abnormalities and skeletal defects, as well as progressive bone marrow failure leading to aplastic anemia typically prior to the patient reaching his or her teens. Patients with FA have a predisposition to multiple malignancies, including leukemia and solid tumors [1]. There are currently 17 FA complementation groups (*FANCA*, *B*, *C*, *D1*, *D2*, *E*, *F*, *G*, *I*, *J*, *L*, *M*, *N*, *O*, *P*, *Q*, and S), each representing a functional gene that can be mutated to cause the disorder. These proteins all appear to work in conjunction with the BRCA proteins to preserve genomic stability through the repair of certain types of DNA damage [2, 3]. FA patient cells are highly sensitive to DNA interstrand crosslinking agents such as mitomycin C, cisplatin, diepoxybutane and melphalan [4].

Following DNA damage the FA core complex, which includes proteins FANCA, B, C, E, F, G, L, and M, is activated and the complex migrates into the nucleus from the cytoplasm [5]. Once inside the nucleus, the activated FA core complex can directly interact with the FANCD2-FANCI protein complex through domains on FANCE [6] and serves as an E3 ligase complex to monoubiquitinate both FANCD2 and FANCI [7, 8]. Monoubiquitinated FANCD2 dissociates from FANCI [9] and binds to damaged regions in the chromatin forming nuclear repair foci in conjunction with BRCA1, BRCA2 (FANCD1), RAD51, and other repair-associated proteins [10–13]. Deficiency in FA core proteins or FANCD2 and FANCI causes sensitivity to DNA crosslinking agents [14–16]. FANCD2 is required for complete activation of DNA replication and damage checkpoints [17], and loss of FANCD2 causes increase in γH2AX levels, indicating the persistence of DNA double strand breaks [17].

FANCJ, also known as the BRCA1-associated C-terminal helicase (BACH1) and the BRCA1-interacting protein (BRIP1), is a 5′-to-3′ DNA helicase that serves as a tumor suppressor and as a mediator of chromosomal stability [18–20]. FANCJ is a member of the DEAH family of helicases and exhibits preference for resolving forked duplex DNA, 5′ flaps, 3-stranded displacement loops (D-loops) [21], DNA triplexes [22], and G-quadruplex structures (G4s) [23, 24].

Evidence from FA-J patient cells, which are deficient in FANCJ activity, has shown that this protein is not required for the monoubiquitination of FANCD2 [25]; therefore, FANCJ has long been considered to function downstream of FANCD2 activation within the FA repair pathway or independent of FANCD2 [25]. However, more recent evidence suggests this may not be the case. Zhang et al showed that FANCJ is necessary for proper FANCD2 foci formation in response to damage caused by a DNA crosslinking agent [26]. FANCJ and FANCD2 have also been shown to directly interact, particularly in undamaged cells. Furthermore, FANCJ modulates FANCD2 association with chromatin in response to DNA damage and FANCD2 reciprocally regulates the formation of FANCJ foci [27].

Much of the work done to elucidate the functions and order of operation of the Fanconi anemia proteins has been done in clinically-relevant FA patient cells. Here, we examined the role of FANCJ by transiently depleting it from cells that are otherwise considered to be normal for the FA repair pathway. We found that in a vast majority of the cell lines, depletion of FANCJ causes the loss of FANCD2 and FANCI proteins. Our studies further demonstrated that in the absence of FANCJ, FANCD2 is targeted for degradation by both the ubiquitin proteasome pathway and a Caspase-3 dependent mechanism. Ectopic complementation of either wild-type FANCJ or a helicase dead (FANCJ-K52R) mutant both efficiently rescued FANCD2/FANCI proteins from degradation, suggesting FANCJ protein, but not its helicase activity, is important for their stability. Taken together the results of this study indicate a new and important role for FANCJ in regulating the stability of FANCD2 in the absence of external DNA damage.

## RESULTS

### Transient down-regulation of FANCJ causes concurrent diminution of FANCD2 and FANCI in normal and multiple cancer cell lines

**Figure 1:**
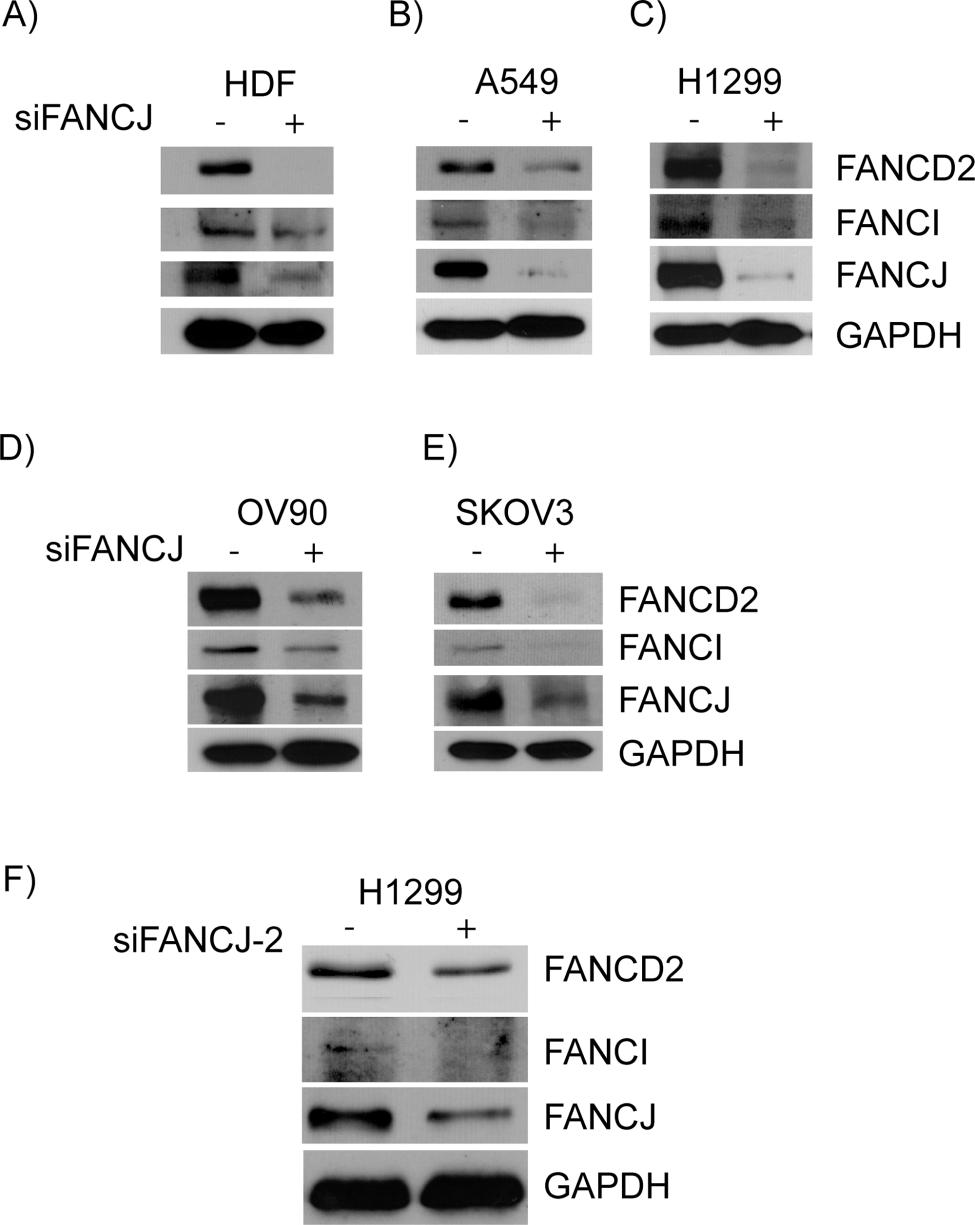
Down-regulation or loss of FANCJ concomitantly diminishes FANCD2 and FANCI proteins in multiple cell lines HDF **A.**, A549 **B.**, H1299 **C.**, OV90 **D.** and SKVO3 **E.** cells were transfected with control or FANCJ siRNAs. After 48 hours whole cell lysates were collected, normalized for total protein concentration, and assessed for the levels of FANCD2, FANCI, FANCJ, and GAPDH proteins by Western blotting. **F.** A second, previously validated, FANCJ siRNA (FANCJ-2) was used to verify that the effects were not the result of non-specific interactions of the original siRNA.

In response to DNA damage, FANCJ is primarily known to act downstream of the FA core complex and the monoubiquitination of FANCD2, which is considered to be activation of FA pathway [25]. Contrarily, there is increased evidence suggesting that FANCJ and FANCD2 directly interact with each other and that FANCJ is necessary for proper FANCD2 response to DNA damage [26, 27]. However, the molecular basis for the interaction between FANCJ and FANCD2 and their regulation in the absence of external DNA damage is not clear. In order to assess the impact of FANCJ status on FANCD2 and its regulatory partner FANCI, FANCJ was transiently down-regulated in a broad spectrum of cell lines that are thought to have an intact complement of FA proteins. Five cell lines were tested including a normal cell line, human dermal fibroblasts (HDF) immortalized with h-TERT. As shown in Figure 1, siRNA mediated down-regulation of FANCJ lead to concomitant loss of FANCD2 and its regulatory partner FANCI in the absence of exposure to any external DNA damage. As cell lines used were originated from different tissue origins, eg., HDF cells are human dermal fibroblasts (1A), A549 (1B) and H1299 (1C) are non-small cell lung cancer cell lines, OV90 (1D) and SKOV3 (1E) are ovarian cancer cell lines, these results suggest FANCJ status is important for the stability or expression of FANCD2 and FANCI proteins in a variety of cell types.

In order to rule out that these FANCJ down-regulation effects on FANCD2 and FANCI stability are due to siRNA-mediated off target effects, a previously validated second siRNA targeting a different sequence of FANCJ was used [48]. Consistent with the results from Figures 1A to 1E, down-regulation of FANCJ by siFANCJ-2 also exhibited concomitant diminution of FANCD2 and FANCI proteins (Figure 1F). These results rule out any possible off target effects of siRNAs and suggest that FANCJ protein is important for the stability or expression of FANCD2 and FANCI proteins.

### FANCJ is important for the stability of FANCD2 protein

**Figure 2:**
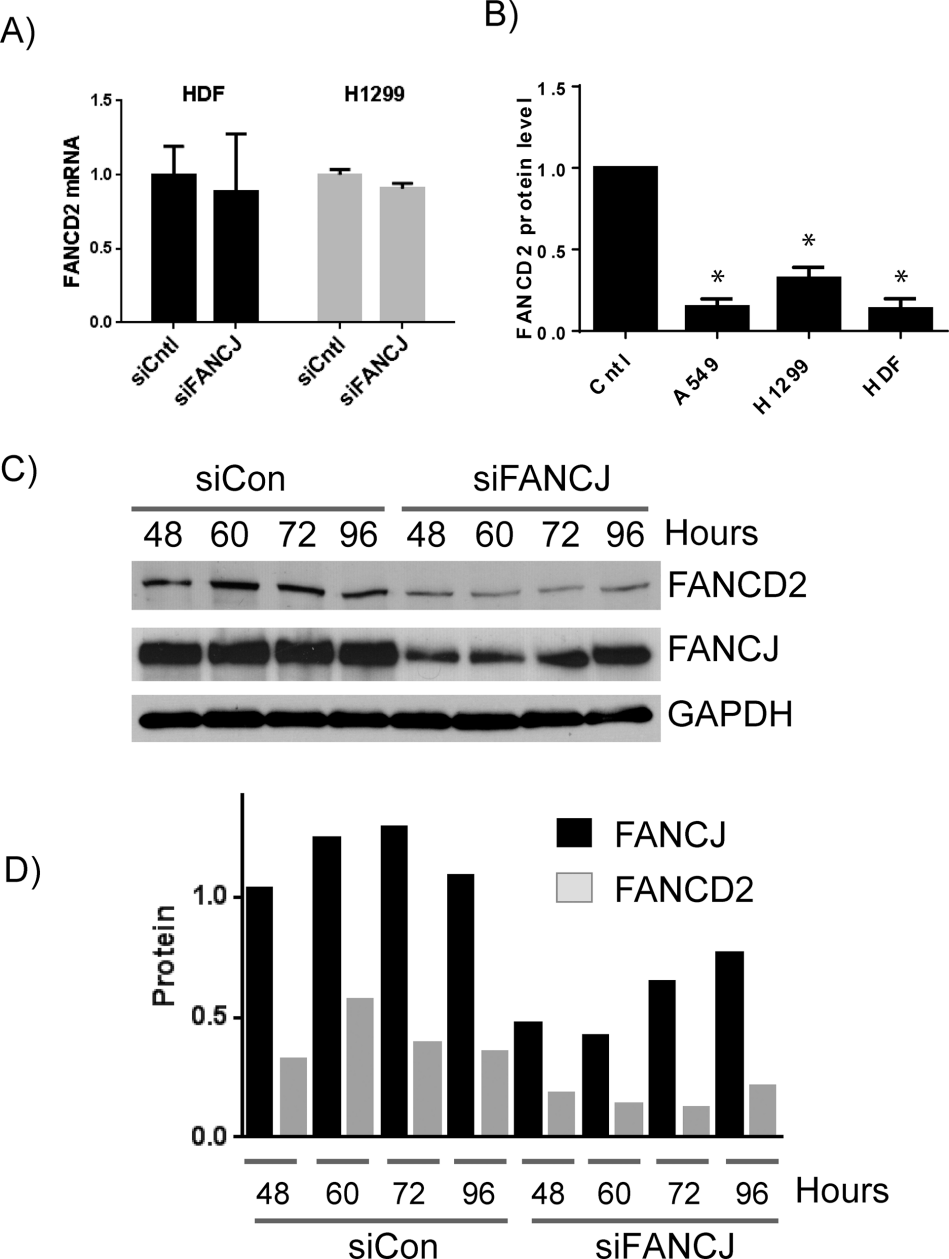
FANCJ regulates FANCD2 concentration at the protein level **A.** Total mRNA was isolated from HDF and H1299 cells transfected with control or FANCJ siRNAs. The levels of FANCD2 mRNA were analyzed in triplicate and normalized to GAPDH message. Error bars represent standard error of the means for all samples. No significant differences were noted between siCntl and siFANCJ samples. **B.** FANCJ was depleted in several cell lines in multiple independent experiments (between 5 and 7 for each cell line) and FANCD2 levels were quantitated by Western blot and averaged. Error bars represent standard error of means of all experiments. * indicates the value is significantly different from control sample (*P* < 0.05). **C.** To determine if FANCD2 levels mirror those of FANCJ or if loss of FANCJ leads to irreparable loss of FANCD2, FANCJ was depleted from H1299 cells by siRNA, and both FANCJ and FANCD2 protein levels were monitored over time (96 hours) until both began to increase. **D.** Densitometry of the time course for 1 representative experiment of three independent samples. FANCJ is represented by black bars and FANCD2 protein by gray.

To determine whether FANCJ status has an effect on protein or transcript level of FANCD2, total mRNA was isolated and subjected to quantitative RT-PCR using FANCD2 primers. As shown in the Figure 2A, no significant differences in FANCD2 mRNA levels were observed in FANCJ-depleted HDF and H1299 cells when compared to control siRNA-transfected cells. Similar results were also observed when tested for FANCI mRNA levels in these cells (data not shown), indicating FANCJ depletion does not significantly affect the transcription of FANCD2 and FANCI genes. Moreover, when average levels of FANCD2 (Figure 2B) and FANCI (data not shown) proteins were calculated from multiple experiments, FANCJ down-regulation caused a several fold decrease in FANCD2 and FANCI in multiple cell lines, and correlated well with the efficiency of FANCJ down-regulation. Taken together these data suggest that FANCJ is important for the stability of FANCD2 and FANCI proteins.

To further determine the reliance of FANCD2 protein stability to FANCJ status, H1299 cells were transiently depleted of FANCJ and subsequently monitored for several days until FANCJ levels returned to near normal levels. FANCJ siRNAs used in this study effectively decrease FANCJ by 48 hours post transfection (Figure 1C and Figure 2C). The effects of FANCJ siRNAs begin to fade after 72 hours of their transfection, where FANCJ protein levels start to increase (Figures 2C and 2D). Moreover, the ratio of FANCD2 to FANCJ is relatively consistent in both control and FANCJ-depleted cells, until FANCJ levels begin to rise at 96 hours (Figure 2D).

**Figure 3:**
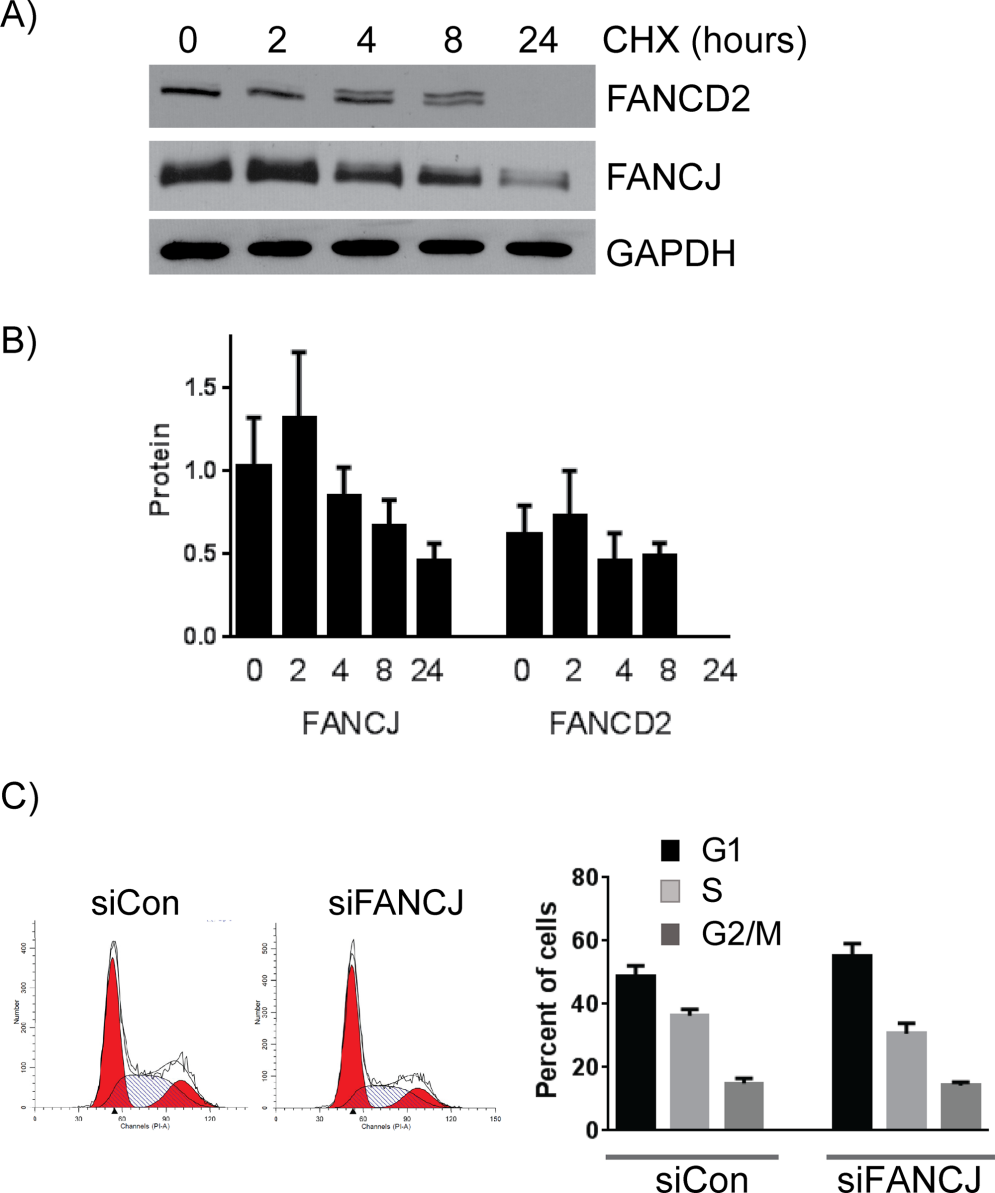
FANCJ protein is more stable than FANCD2 and does not significantly alter the cell cycle profile **A.** Long term FANCJ and FANCD2 protein stability was measured by treating H1299 cells with the protein biosynthesis inhibitor cycloheximide. **B.** Summary of three experiments of cycloheximide treatment in H1299. Error bars represent standard error of means for all experiments. **C.** H1299 cells were treated with control or FANCJ siRNAs, grown for 48 hours and treated with propidium iodide. The impact of knocking down FANCJ on the cell cycle was assessed by FACS. One representative, of multiple, cell cycle profile is shown. The graph represents the average values for cell cycle distribution from at least 3independent experiments. There were no statistical differences between the distribution of the control and FANCJ-depleted samples.

To further study the stabilities of these proteins in cells, H1299 cells were treated with protein synthesis inhibitor cycloheximide and the levels of both FANCD2 and FANCJ were assessed. As shown in the Figures 3A and 3B, levels of both the proteins started decreasing with time upon cycloheximide treatment. Consistent with the above observations, FANCJ was found to have a longer half-life than FANCD2 in cells (Figure 3B). These results provide further evidence that the stability of FANCD2 is directly tied to FANCJ in these cells.

FANCJ has been shown to be an important regulator of cell cycle progression checkpoints. In order to determine whether FANCJ is causing the diminished FANCD2 and FANCI levels as a consequence of cell cycle arrest, cell cycle profiles of H1299 cells transfected with either control or FANCJ siRNA were compared. As shown in Figure 3C, there is only a 5 to 7% decrease in S-phase cells observed in FANCJ-depleted cells when compared with control cells. Such a small reduction in the S-phase population should not cause the dramatic (more than 5-fold) decrease in FANCD2 and FANCI proteins observed in these cells. Similarly, no significant differences were observed in actively replicating cells, when fractions of BrdU incorporating cells were assessed (data not shown). These data exclude cell cycle effects as an important determinant for FANCD2 and FANCI stability in FANCJ-depleted cells.

### Transient knockdown of FANCD2 does not cause substantial decrease in FANCJ protein

**Figure 4:**
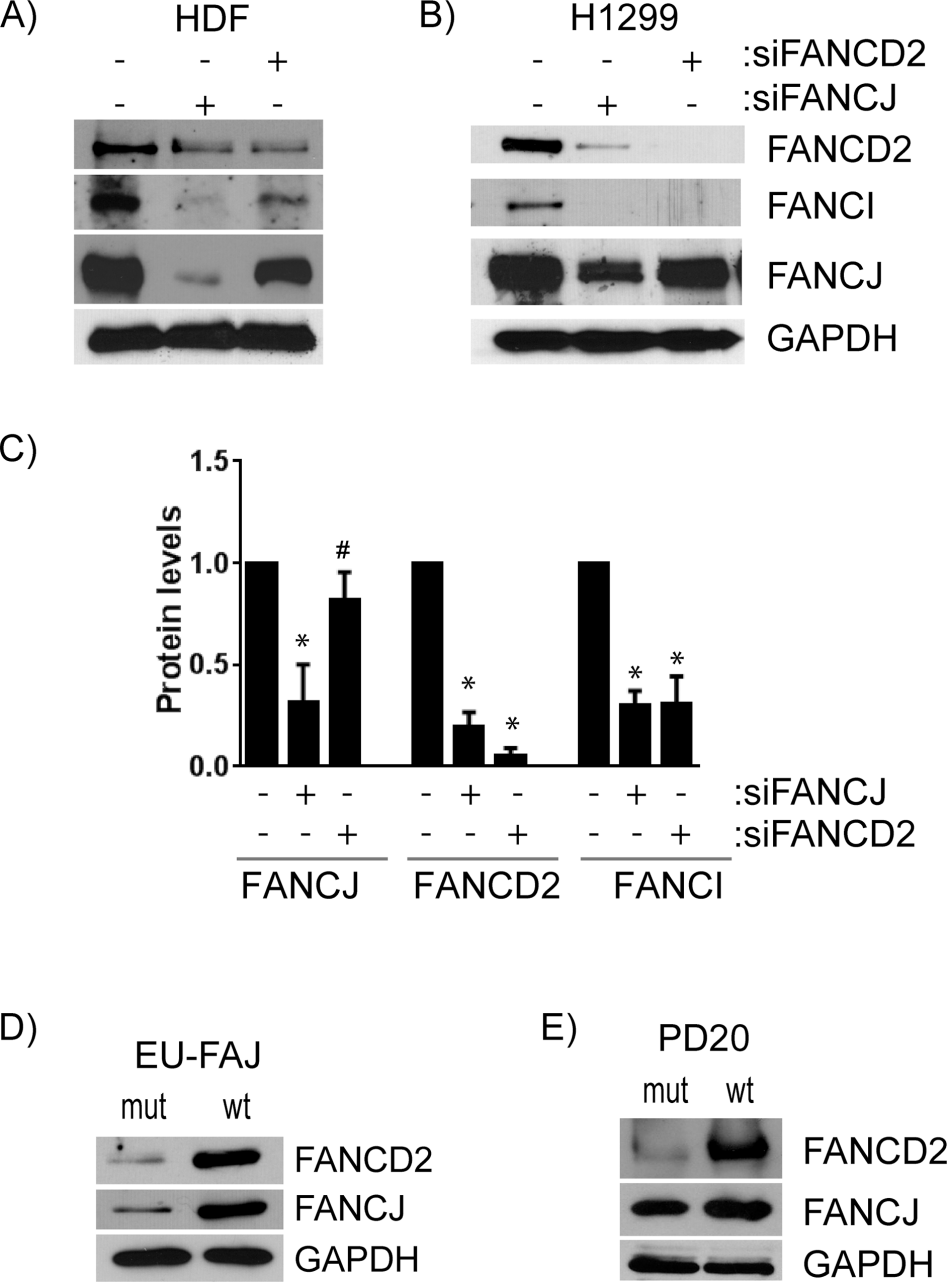
FANCJ regulates FANCD2 stability, but FANCD2 has little to no effect on FANCJ protein levels **A.** HDF or **B.** H1299 cells were transfected with siRNAs for either FANCJ or FANCD2 and the levels of FANCJ, FANCD2, and FANCI were measured by Western blot. **C.** The average values of FANCJ, FANCD2, and FANCI in cells treated with siRNAs for FANCJ or FANCD2, from at least three independent experiments, were normalized to the levels in H1299 cells treated with control siRNA. Bars represent standard error from multiple (3–4) independent experiments. * indicates the value is significantly altered from control (*P* < 0.05). # indicates that FANCJ levels are significantly different between cells treated with siFANCJ and siFANCD2 but not changed from control (*p* < 0.05) indicating that depletion of FANCD2 does not substantially alter FANCJ levels. **D.** FANCJ-deficient EUFA30 and the FANCJ-corrected counterpart cell line were examined by Western blot for expression of FANCD2. **E.** FANCD2-deficient PD20 and its FANCD2-corrected variant were analyzed by immunoblotting for FANCJ.

FANCD2 protein levels appear to frequently be tied to those of FANCJ. To assess whether the converse is also true, FANCJ and FANCD2 were each transiently down-regulated and their impact on each other as well as FANCI levels were measured. Consistent with the results in Figure 1, FANCJ depletion drastically reduced FANCD2 (average 80% decrease) and FANCI (65% decrease) protein levels. Contrarily, knocking down FANCD2 caused only a modest decrease in FANCJ protein levels in both HDF (36% decrease; Figure 4A) and H1299 (24% depletion; Figure 4B) cell lines. Therefore, FANCJ is necessary for proper stabilization of FANCD2, but FANCD2 exerts little effect on FANCJ. However, depletion of either FANCJ or FANCD2 caused substantial loss of FANCI protein in all the cell lines (Figures 4A, 4B, 4C). In H1299 cells, both FANCJ and FANCD2 knockdown had very similar impacts on FANCI, with 65% and 61% decrease, respectively (Figure 4B & 4C).

To further confirm that FANCJ protein is important for FANCD2 and FANCI protein stability, FANCJ-deficient cell line isolated from FA patient (EUFA30) and its wild-type FANCJ-corrected counterpart were assessed for FANCD2. As shown in Figure 4D, the level of FANCD2 protein was almost undetectable compared to the wild-type FANCJ-corrected cells. To further probe FANCD2 status effects on FANCJ protein, FANCD2 mutant cells (PD20) were compared with their wild-type FANCD2-corrected cells. Consistent with the data from transient siRNA knockdown, sustained FANCD2 deficiency minimally affected FANCJ protein levels (Figure 4E).

### FANCD2 degradation in FANCJ-depleted cells occurs through both ubiquitin proteasome and caspase pathways

**Figure 5:**
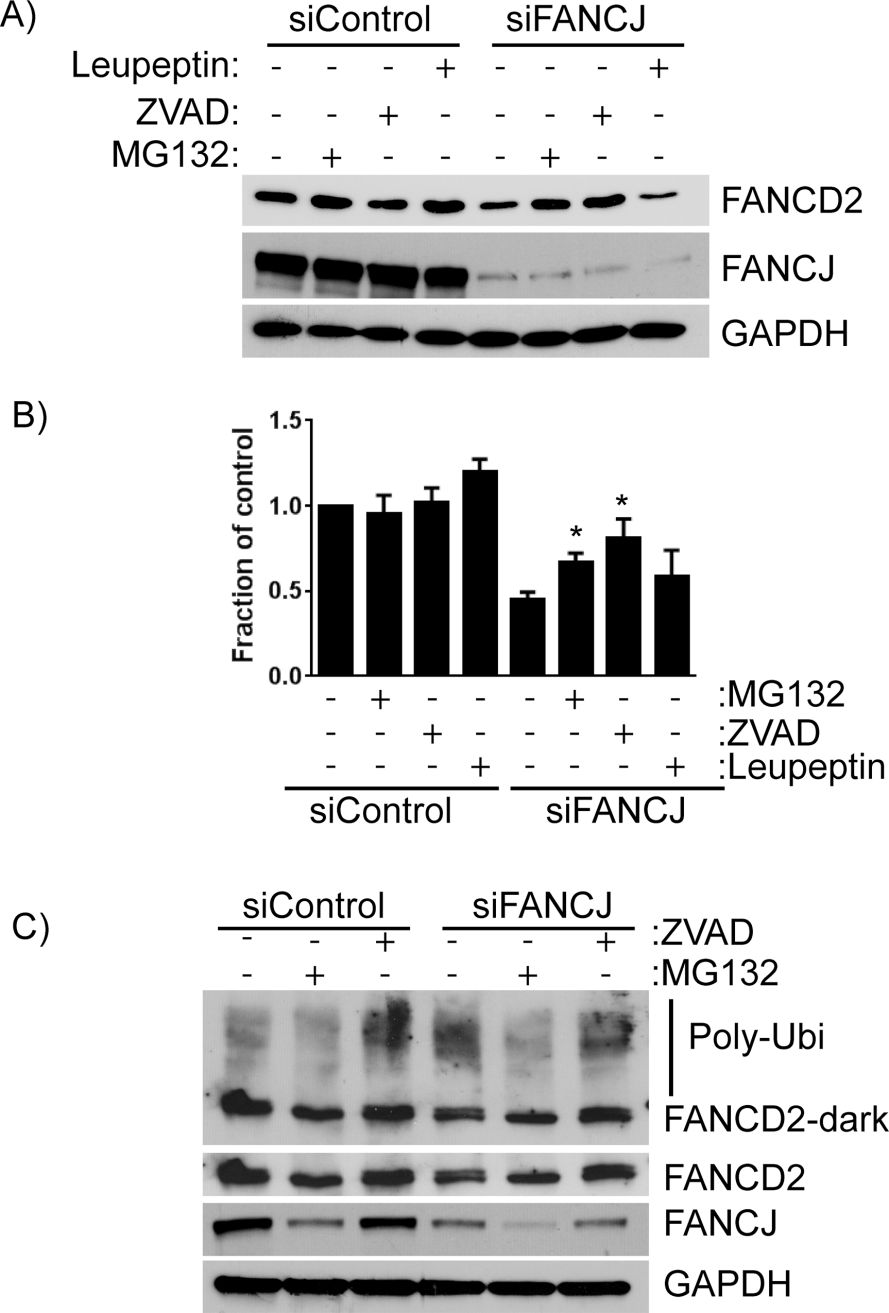
FANCD2 degradation in the absence of FANCJ is dependent on both the ubiquitin proteasome pathway and caspases **A.** H1299 cells treated with control or FANCJ siRNAs were exposed to several different protease inhibitors - leupeptin (10 μM), MG-132 (3 μM), or Z-VAD-FMK (10 μM) for 12 hours prior to harvesting. Total cellular proteins were extracted and FANCD2 levels measured with Western blotting. **B.** Average levels of FANCD2 in control and FANCJ-depleted cells for at least 5 separate experiments were measured by densitometry and normalized to GAPDH. * Represents the values are significantly different from the corresponding vehicle control (*p* < 0.05). **C.** The impact of the ubiquitin proteasome inhibitor MG-132 and the caspase inhibitor Z-VAD-FMK were confirmed in the normal, immortalized HDF cell line. Stabilization of high molecular weight FANCD2 (polyubiquitinated) were observed in Z-VAD-FMK treated cells. Light and dark exposures of FANCD2 are shown to more clearly indicate the changes in unmodified, monoubiquitinated, and polyubiquitinated bands.

FANCJ clearly has a role in regulating FANCD2 protein levels, though it does not alter transcription of its gene. To determine the mechanism by which FANCJ controls FANCD2 stability, various protease inhibitors were administered to FANCJ-depleted cells. The inhibitors used include leupeptin, which inhibits many of the common proteases found in lysosomes, MG-132, which blocks the ubiquitin proteasome, and Z-VAD-FMK, a caspase inhibitor. Caspases are not commonly considered proteases that regulate protein stability in non-apoptotic cells; however, there is recent evidence suggesting that they can regulate FANCD2 protein stability [28]. Surprisingly, it appears that FANCJ regulates FANCD2 stability through 2 separate pathways, as both MG-132 and Z-VAD-FMK restored FANCD2 levels to near normal levels in H1299 lung cancer cells (84% and 93% of normal, respectively; Figure 5A and 5B). On the other hand, leupeptin treatment had no effect on FANCD2 levels. To confirm that these effects are not specific to H1299 cells, HDF cells were transfected with appropriate siRNAs and treated with MG-132 and Z-VAD-FMK. Unexpectedly, MG-132 treatment caused a drop in FANCJ protein levels of control siRNA-transfected cells (Figure 5C) and this was accompanied by a decrease in FANCD2 in these cells. However, in the FANCJ-depleted cells there was approximately 50% restoration of FANCD2 protein, suggesting a role for ubiquitin proteasomal system in regulation of FANCD2 in the absence of FANCJ. Interestingly, exposure to MG-132 caused loss of both the mono- and polyubiquitinated forms of FANCD2 which are present in the other samples (DMSO and Z-VAD-FMK) (Figure 5C), suggesting that in these cells MG-132 may be causing rapid depletion of the ubiquitin pool. Irrespectively, consistent with the H1299 cell data, caspase inhibitor (Z-VAD-FMK) effectively restored FANCD2 levels in FANCJ-depleted cells. Likewise, the levels of monoubiquitinated and high molecular weight FANCD2 (poly-ubiquitinated) were increased in Z-VAD-FMK treated cells (Figure 5C), suggesting an important role for caspases in regulation of FANCD2. While it is unusual for proteins to be degraded by multiple pathways, there is precedent for a protein to be degraded by both caspase- and proteasome-dependent pathways [29]. This is very similar to what appears to be happening with FANCD2.

### FANCJ directly interacts with FANCD2

**Figure 6:**
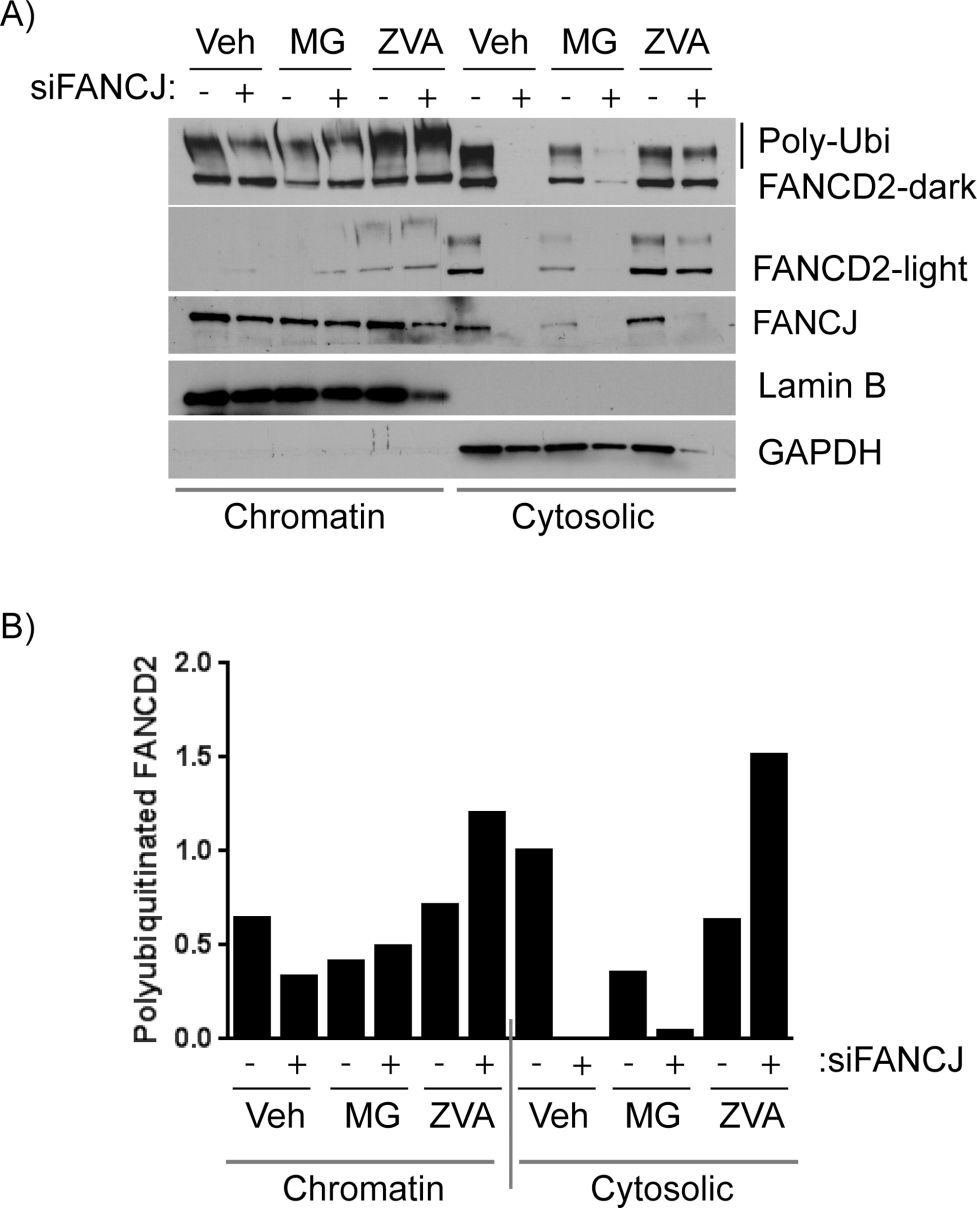
FANCJ deficiency compromises the stability of FANCD2 in both cytoplasmic and chromatin fractions **A.** H1299 cells were transfected with control or FANCJ siRNAs, treated with the DMSO (Veh, vehicle) or ubiquitin proteasome inhibitor MG-132 (3 μM) or the panCaspase inhibitor Z-VAD-FMK (10 μM) for 12 hours prior to harvesting. Cells were then fractionated to separate cytoplasmic and chromatin-associated proteins. These protein fractions were examined by Western blot to analyze the effect of the protease inhibitors on FANCD2 degradation. Stabilization of high molecular weight FANCD2 (polyubiquitinated) in both chromatin and cytoplasmic fractions were observed in cells treated with Z-VAD-FMK. Lamin B1 and GAPDH serve as controls for efficient separation of chromatin-associated and cytoplasmic proteins, respectively. **B.** The large polyubiquitinated (from dark exposure) FANCD2 bands were quantitated and normalized to Lamin B1 for chromatin-associated extracts and GAPDH for cytoplasmic proteins. The quantity of each protein in the vehicle-treated control sample was nominally set to 1 to allow easier comparison.

Recent studies have shown that FANCJ and FANCD2 interact with each other, especially in undamaged cells [27]. This interaction was confirmed in several of the cell lines used in this study (data not shown). To further characterize these interactions in cells that are not exposed to external DNA damage, the proteins from H1299 cells were fractioned into cytosolic and chromatin components. While both FANCJ and FANCD2 proteins were largely confined to the cytosolic fraction (data not shown and Figure 6). Consistent with the previous studies, FANCJ and FANCD2 co-immunoprecipitated each other, suggesting these proteins interact in both of the cellular compartments (data not shown).

To further assess the role of ubiquitin-mediated proteasome system and caspases in regulation of FANCD2 with respect to FANCJ status, H1299 cells were transfected with siRNAs and exposed to MG-132 and Z-VAD-FMK, and the fractionated cell lysates were examined. Interestingly, when FANCJ is transiently depleted, FANCD2 levels in the cytosolic fraction, where most of the interaction occurs, drops precipitously. While the levels of these proteins in the chromatin fractions are very low compared to the cytoplasmic fractions, the FANCJ depletion effects also correlated in the chromatin-bound FANCD2 (Figure 6). Moreover, several-fold increase in high molecular weight (poly-ubiquitinated) FANCD2 bands was observed in pan-caspase inhibitor Z-VAD-FMK treated cells (Figure 6). Curiously, the ubiquitin proteasome inhibitor MG-132 causes a decrease in FANCJ in the nucleus of these cells which also causes a drop in basal FANCD2; however, it does appear to somewhat protect degradation of FANCD2 when FANCJ is depleted. These data strongly implicate FANCJ as being necessary for proper stabilization of FANCD2 in both cytoplasm and nuclei of undamaged cells. However, the effects of MG-132 on FANCJ need to be further studied to understand the differential responses.

### Caspase-3 degrades FANCD2 in the absence of FANCJ

**Figure 7:**
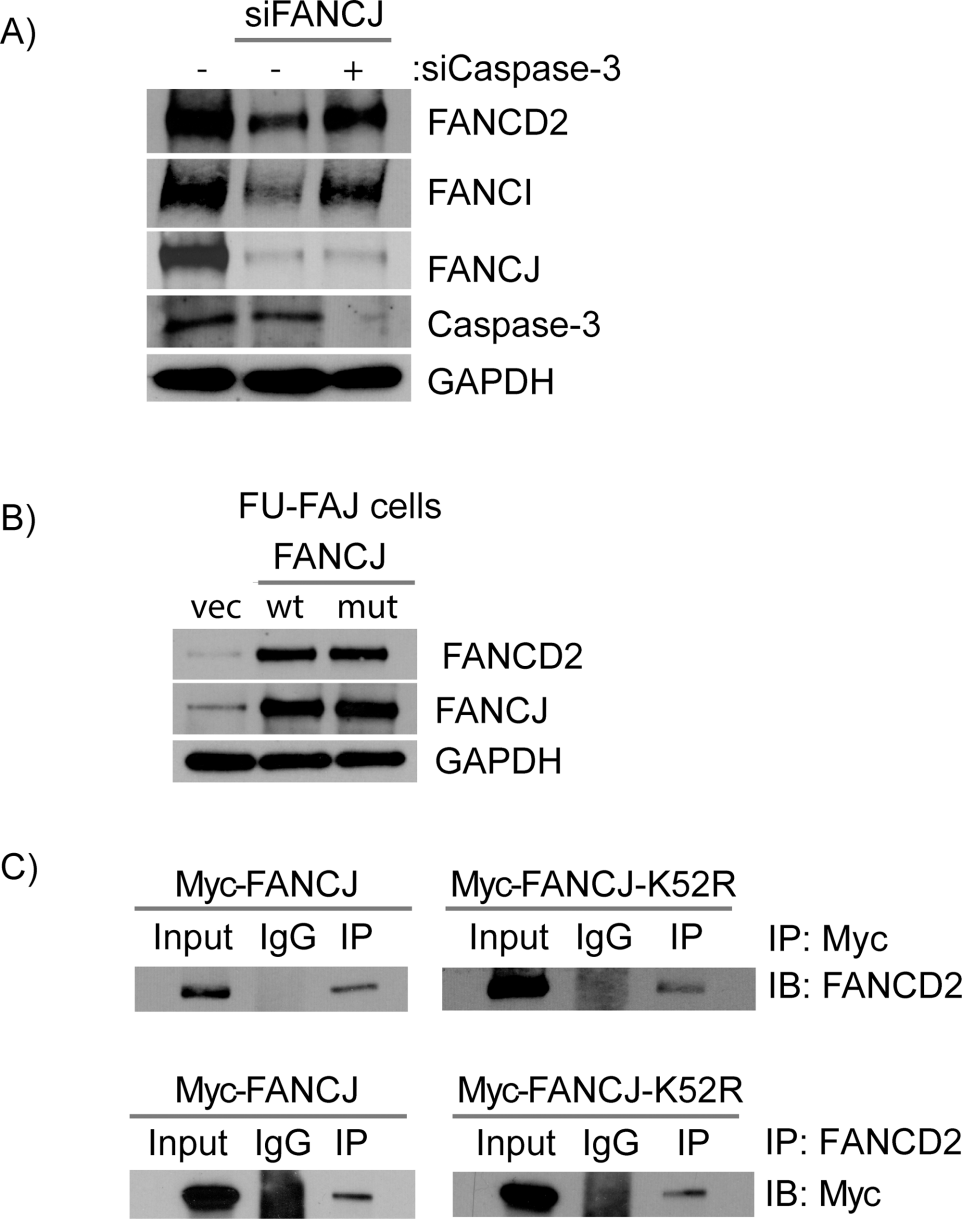
Caspase-3 degrades FANCD2 in the absence of FANCJ and FANCJ helicase activity is not required for the stabilization of FANCD2 **A.** H1299 cells were transfected with control, FANCJ, and Caspase-3 siRNAs as indicated, and FANCD2 and FANCI proteins were examined by Western blots. **B.** The FANCJ-defective patient cell line EUFA30 was transfected with plasmids containing either wild-type or the helicase defective mutant (K52R) FANCJ. The effects of these two FANCJ variants on FANCD2 stability were determined by Western blot analysis. **C.** Protein extracts from H1299 cells expressing either a wild-type or helicase-defective mutant (K52R) FANCJ (both labeled with a Myc-tag) were selected to co-immunoprecipitation with antibodies directed against either FANCD2 or the Myc-tag on the 2 FANCJ variants (WT and K52R). The precipitated proteins were blotted for the corresponding partner protein (FANCD2 or Myc). FANCD2 was able to pull down both wild-type and the helicase-deficient FANCJ and FANCD2 in turn were found in immunoprecipations of both FANCJs.

Caspase-3 has previously been shown to be capable of degrading FANCD2 in damaged and apoptotic cells [28]. The pan-caspase inhibitor Z-VAD-FMK prevented loss of FANCD2 in response to FANCJ depletion; therefore, one or more caspases were capable of acting in undamaged, non-apoptotic, normal cells to degrade FANCD2. To determine, if caspase-3 was responsible for degrading FANCD2 in FANCJ-deficient cells, it was co-depleted with FANCJ using siRNAs and the levels of FANCD2 and FANCI were monitored. Remarkably, knocking down caspase-3 in FANCJ down-regulated cells completely restored FANCD2 and FANCI levels to normal (Figure 7A). These data confirm that caspase-3 is the major player in regulating FANCD2 and FANCI proteins in the absence of FANCJ.

### FANCJ protein, but not its helicase activity, is required for FANCD2 stability

In order to further elucidate how FANCJ regulates FANCD2 stability, FANCJ-deficient mutant cells were transfected with plasmids expressing either wild-type or a helicase-deficient mutant (K52R) FANCJ. Interestingly, expression of either wild-type or helicase-defective mutant FANCJ equally restored FANCD2 and FANCI protein levels (Figure 7B). These data confirm that FANCJ helicase activity is dispensable, but the intact FANCJ protein is important for the stability of FANCD2 and FANCI proteins. Furthermore, helicase dead FANCJ (K52R) binds to FANCD2 and immunoprecipitates with the same efficiency as wild-type FANCJ (Figure 7C). These data identify novel regulatory functions of FANCJ apart from its role in resolving atypical DNA structures. Nevertheless, there are many different FANCJ mutations that have been identified in FA patients as well as in different cancers and it is possible that some of these may alter FANCJ and FANCD2 interactions. The detection and characterization of any such mutations and their biological consequences would be of great interest in light of the data presented here.

### Depletion of FANCJ causes decreased activation of the DDR

**Figure 8:**
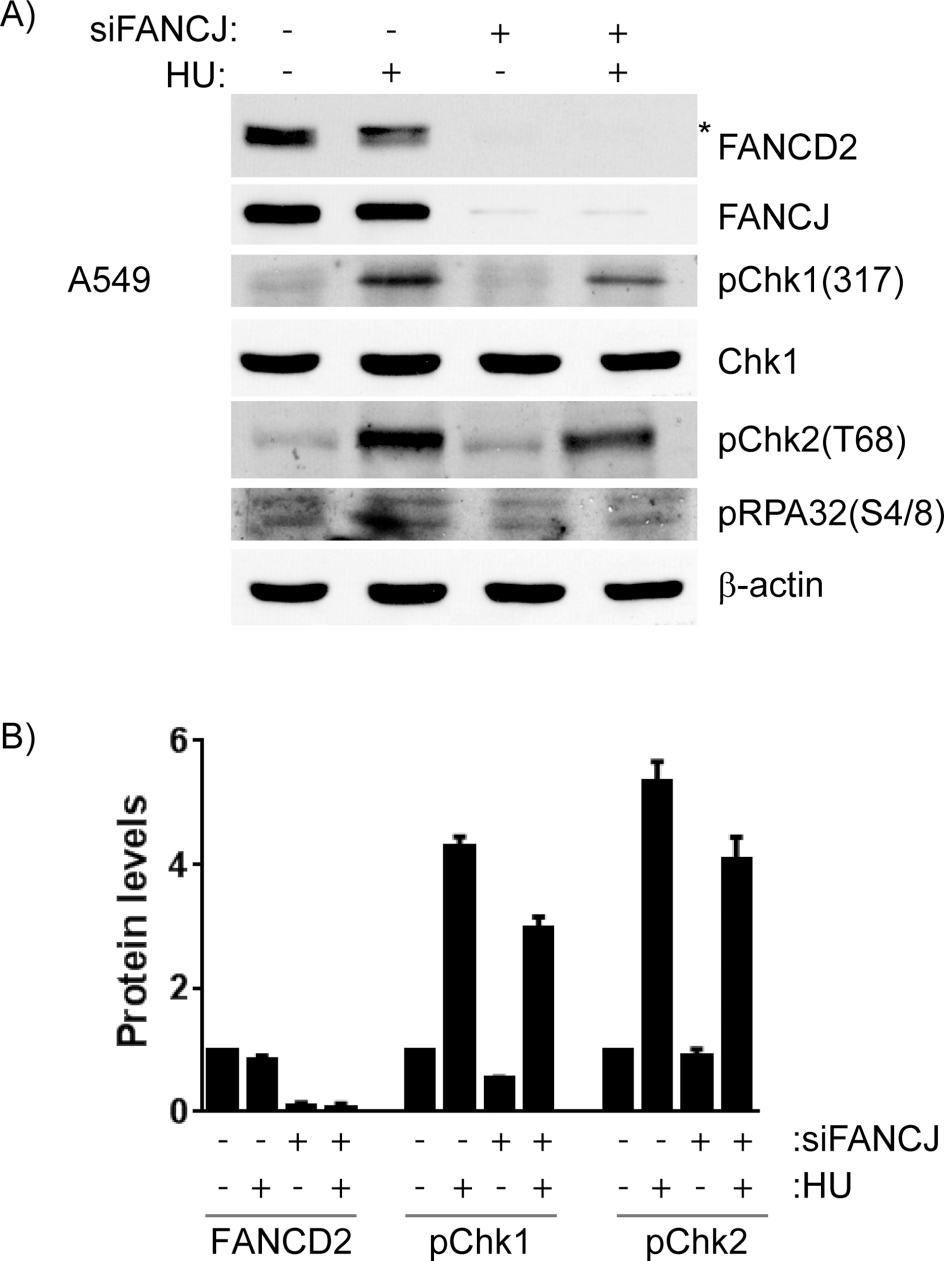
Cells lacking FANCJ are unable to properly respond to DNA damage caused by stalled replication forks A549 cells were transfected with control or FANCJ siRNAs twice with 24 hours interval. After 48 hours post-transfection, cells were treated with DMSO or Hydroxyurea (2 mM) for 4 hours. **A.** Whole cell lysates were assessed for FANCD2 and indicated DDR proteins by Western blot analysis. * indicates monoubiquitinated form of FANCD2. **B.** Protein levels of FANCD2 and activated DNA damage checkpoint kinases, Chk1 (S317) and Chk2 (T68) were measured after normalizing to β-actin, in control and FANCJ-depleted cells. Error bars represent the standard error of 3 independent experiments. Hydroxyurea treatment caused significant increases in phosphorylation of both Chk1 and Chk2, and both were substantially diminished by FANCJ depletion.

FANCD2 is important for the proper response to the DNA damage induced by the replication blocker hydroxyurea (HU). In HU treated cells FANCD2 interaction with RAD51 and RAD18 increases and this is necessary for proper PCNA activation. Interestingly, unlike in response to DNA crosslinking agents, FANCD2 monoubiquitination is not required [30]. In order to determine if the decrease in FANCD2 in FANCJ-depleted cells, could be alleviated by HU-induced replication stress, control and FANCJ down-regulated cells were treated with HU and monitored for early DDR, FANCD2 monoubiquitination and protein levels. As shown in the Figure 8, HU treatment induced replication stress-mediated DDR, as evidenced by activation of checkpoint proteins Chk1 and Chk2 and phosphorylation of RPA and monoubiquitination of FANCD2 in siControl cells. However, the levels of FANCD2 remain low in FANCJ-depleted cells even when treated with HU. Similar decrease in FANCD2 nuclear foci were also observed in FANCJ depleted cells in response to HU (data not shown). Consistent with the previous reports, FANCJ deficiency also attenuated early DDR such as phosphorylation of checkpoint kinases Chk1, Chk2 and single strand DNA binding protein RPA [31, 32]. Therefore, our data suggests that FANCJ is important for early DDR to replication blocks induced by HU, and in the absence of FANCJ, DDR has little affect on FANCD2/FANCI stability.

## DISCUSSION

The DNA helicase FANCJ has long been considered to function downstream of FANCD2 in the cellular response to several types of DNA damage, including DNA crosslinks, stalled or collapsed replication forks and strand breaks [33, 34]. This conclusion is based on a multitude of findings showing that in FA patient cell lines devoid of functional FANCJ; FANCD2 is still monoubiquitinated in response to DNA damage and still forms foci on damaged DNA along with other DDR proteins. Contrary to the placement of FANCJ late in the DNA repair process, there is evidence FANCJ can sense DNA damage [35], is associated with the DNA replication fork and works to prevent fork stalling during replication stress events as well as resolving certain DNA secondary structures, such as the G-quadruplex, that might inhibit replication [36, 37]. Furthermore, FANCJ and FANCD2 form a complex in response to multiple types of DNA damaging agents and their nuclear foci were found to colocalize at damaged DNA [26]. FANCD2 foci formation was found to be more efficient in FANCJ-corrected versus uncorrected FA-J cells (EUFA30) [26]. Additionally, FANCD2 and FANCJ were found to be necessary for the efficient foci formation in response to the DNA crosslinking agent mitomycin C [27]. There is still much yet to be discovered regarding how the proteins of the FA DNA repair pathway function. In fact new proteins are still being assigned to the FA family [38]. Recently, it has also been shown that FANCD2 does not have to be monoubiquitinated in order to function in response to at least some types of DNA damage [30], a finding that opposes the long-held model of the DDR.

This study shows that FANCJ is required to stabilize FANCD2 protein in cells with normal, background levels of DNA damage. When FANCJ is transiently knocked down with siRNA in a variety of cell lines, there is a concomitant, drastic decrease in FANCD2 levels (Figures 1 & 2B). A FA-J patient cell line, that does not have intact FANCJ also exhibited lower FANCD2 levels than its FANCJ-corrected counterpart (Figure 4D). Interestingly, even though this effect was seen in a variety of cell lines, including both, immortalized, normal cells and cancer cells, this effect was not seen in the breast cancer cell line MCF-7 (data not shown). This suggests that there is some degree of cell line specificity to FANCJ's stabilizing effect. What causes MCF-7 to behave differently is yet to be determined; however, many cancer cells have been shown to have defective or altered DNA repair pathways. The variability shown here emphasizes the need to use multiple cell lines for these types of study.

The FACS analysis of control and FANCJ down-regulated cells showed FANCJ effect on FANCD2 levels was not due to its capacity to regulate the cell cycle (Figure 3C). Similarly, FANCD2 mRNA levels were unaffected by depletion of FANCJ (Figure 2A). Inhibitors of various protease inhibitors were tested to determine the pathway through which FANCD2 is being degraded in the absence of FANCJ. Two very different pathways were found to be responsible. Both the ubiquitin proteasome inhibitor MG-132 and the pan-caspase inhibitor Z-VAD-FMK were able to stabilize FANCD2 with similar efficiencies. Sakai and Sugasawa demonstrated that in damaged and apoptotic cells, caspase-3 could degrade FANCD2 [28]. When caspase-3 was down-regulated by siRNA, FANCD2 returned to near normal levels (Figure 7A). While caspase-3 degrades proteins in apoptotic cells, there is a lack of cleaved caspase-3 in FANCJ-depleted cells, indicating a lack of activation under these conditions. Moreover, we did not observe any significant differences in activation of DNA damage checkpoint kinases Chk1, Chk2 and RPA phosphorylation in FANCJ down-regulated cells when compared to control cells in undamaged conditions (data not shown and Figure 8A). This rules out any influence of DNA damage mediated apoptosis in FANCJ deficient cells. Therefore, the mechanism through which caspase-3 is responsible for FANCD2 degradation needs to be further studied. However, there has recently been some intriguing work showing that cells can maintain a sub-lethal level of activated caspase-3, that activation can be related to DNA damage, and the caspase-3 is capable of proteolytic activity, and can promote genetic instability [39]. More study into this area is warranted.

**Figure 9:**
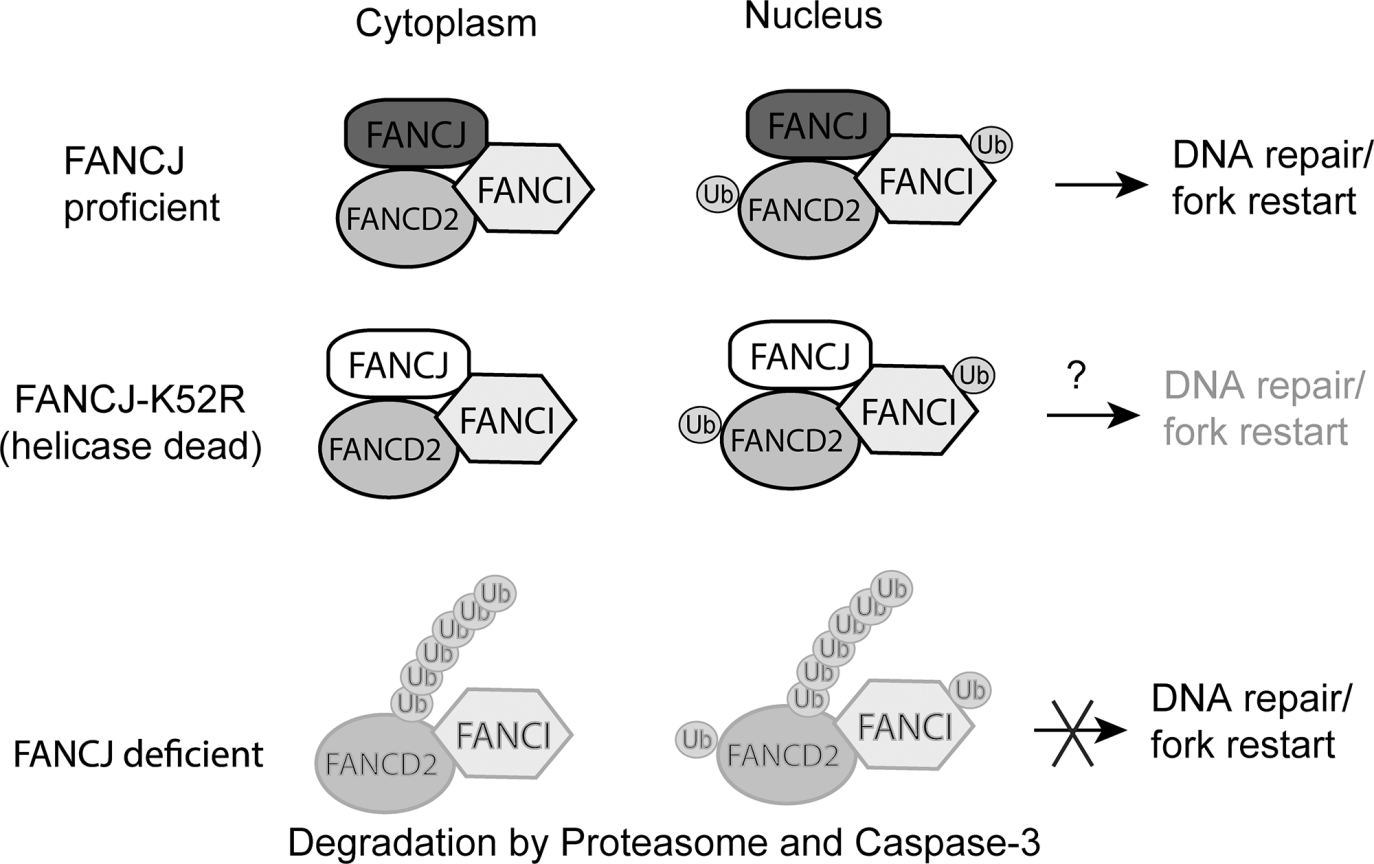
Proposed model for FANCJ stabilization of FANCD2 and FANCI FANCJ exists in a complex with both FANCD2 and FANCI in undamaged cells. This complex exists principally in the cytoplasm, but is also present in the nucleus. When there is DNA damage FANCD2 can be activated by the FA core complex and transmitted to the nucleus to participate in the formation of repair foci at the site of damage. When the K52R helicase-dead mutant, and potentially other FANCJ point mutations, is part of the complex it would protect FANCD2 but may still result in decreased DNA repair. For example, the K52R mutation has previously been shown to increase sensitivity to certain types of DNA damage, such as ionizing radiation [48], suggesting FANCJ helicase functions could be independent from stabilizing FANCD2/FANI proteins. When FANCJ is lost the unprotected FANCD2 and FANCI proteins are degraded by the ubiquitin proteasome and caspase-3.

FANCJ and FANCD2 interact with each other and the majority of the interaction occurs in the cytoplasm, as most of these proteins reside in the cytoplasm in undamaged cells (Figure 6A). This suggests that FANCJ sequesters FANCD2 away from the DNA in undamaged cells. This interaction does not require FANCJ to be capable of hydrolyzing ATP or having its DNA helicase activity. The K52R FANCJ mutant could interact with FANCD2 and protected it from degradation as efficiently as wild-type protein (Figure 7C). The K52R FANCJ has no detectable ATP hydrolysis activity or DNA helicase activity [40] and was more susceptible to certain types of DNA damage and caused a delay in repair of DSBs [40]. The physical interaction between FANCJ and FANCD2 protects the latter protein from degradation through the ubiquitin proteasome and caspase-3, maintaining the protein in a state of readiness to be activated in response to DNA damage (Figure 9). Since the interaction is needed to stabilize FANCD2 in the absence of DNA damage the proteins are kept away from the DNA, potentially explaining why FANCJ's DNA helicase function is dispensable. The precise reason that FANCD2 stability is coupled to FANCJ is of interest and needs to be determined. Interestingly, however, the reverse is not the case, as knocking down FANCD2 had very little effect on FANCJ protein levels (Figure 4A, 4B, 4C, & 4E). FANCJ has been shown to interact with numerous DNA repair proteins, including BRCA1 [41] the BLM helicase [36], Replication protein A (RPA) [42], the nuclease MRE11 [43], MLH1 [44] and TopBP1 [31]. A detailed study needs to be made to determine which of these proteins might also be contained in the complex between FANCJ and FANCD2, or whether these interactions regulate each other through shared binding sequences. For instance, MRE11 and BRCA1 both bind to the same region of FANCJ and it has been speculated that they might compete with each other in binding [43].

FANCJ has been shown to act in a multitude of DDR pathways, and there is evidence that it has functions early in the DNA damage response, and sometimes works independently of the other FA proteins. As a DNA helicase, FANCJ associates with the transcription machinery and works to resolve various DNA secondary structures that occur both spontaneously and as a result of DNA repair processes [24, 37, 41, 45]. FANCJ also interacts directly with the MRN complex (MRE11, RAD50, and NBS1) through binding to MRE11 [43]. The MRN complex is part of the very early response to DNA double strand breaks (DSBs) and recruits FANCJ to the site of the break. The recruited FANCJ in turn regulates the nuclease activity of MRE11 to process the DNA around the break for initiation of repair [43]. Further evidence for an early role of FANCJ in DSB repair is that FANCJ is necessary for the proper recruitment of the BLM helicase to strand breaks. BLM is required for early processing of DSBs for repair [46]. In response to DNA interstrand crosslinks (ICLs), MRE11 does not recruit FANCJ to the damaged DNA. Instead FANCJ recruitment occurs later in the repair process and is dependent upon MLH1 and the FA core complex [43, 44]. These different patterns and times of recruitment of FANCJ to DNA damage, combined with the results of this current study, suggest the possibility that when FANCJ is recruited for a repair pathway independent of the other FA proteins, FANCD2 is left unprotected and is degraded by the cell. However, in a repair pathway that includes FANCD2 and the other FA proteins, such as in ICL repair, FANCD2 is recruited to the damaged DNA before or simultaneously with FANCJ and thus remains protected from degradation. This correlates with the recent finding that the interaction of FANCJ and FANCD2 decreases in a damaged cell [27].

## METHODS

### Cell culture and reagents

The cell lines H1299, A549, MCF7, OV90, and SKOV3 were obtained from ATCC (Manassas, Virginia) and grown according to their instructions, typically in Dulbecco's Modification of Eagle's Medium (DMEM) (Mediatech, Manassas, Virginia) supplemented with 10% fetal bovine serum (FBS) (Omega Scientific, Tarzana, Ca) and 1x Penicillin/Streptomycin (Gibco). Immortalized, non-transformed Human Dermal Fibroblasts (HDF) were cultured as described previously [47]. EUFA30 immortalized fibroblasts and EUFA30FJ, the FANCJ-corrected counterpart were cultured in DMEM with 20% FBS and Pen/Strep. FANCD2-deficient PD20 cells and those corrected with wild-type FANCD2 (supplied by the Fanconi Anemia Research Fund) were grown as described previously [13]. pcDNA3-myc-his-BACH1 WT (Addgene plasmid # 17642) and pcDNA3-myc-his-BACH1 K52R (Addgene plasmid # 17643) plasmids were generous gift from Dr. Ronny Drapkin and have been described [18]. Cycloheximide, Leupeptin, and calpain inhibitor I were purchased from Sigma-Aldrich, MG132 and Z-VAD-FMK were from Selleckchem.

### siRNAs and transfection

To knock down expression of each gene, the following siRNA sequences were used: control AGUUACUCAGCCAAGAACGAUU, FANCJ#1 GUACAGUACCCCACCUUAUUU [20], FANCJ#2 AAACAGCAAGCAACAUUGUUU [48], FANCD2 GCACCGUAUUCAAGUACAAUU [47], and caspase-3 CCCUGGACAACAGUUAUAA. All siRNAs were purchased from Dharmacon. Transfections of siRNA oligonucleotides were done using Lipofectamine 2000 (Invitrogen) following the manufacturer's protocol. For all cell lines, except H1299, cells were transfected twice, 24 hours apart, with siRNA to get more efficient knockdown. One transfection provided sufficient knockdown of target proteins in H1299 cells.

### Western blotting and antibodies

Cells were treated as indicated, washed with cold PBS, and lysed in ice-cold cytoskeletal (CSK) buffer (10 mM PIPES (pH 6.8), 100 mM NaCl, 300 mM sucrose, 3 mM MgCl2, 1 mM EGTA, 1 mM dithiothreitol, 0.1 mM ATP, 1 mM Na3VO4, 10 mM NaF and 0.1% Triton X-100) freshly supplemented with protease and phosphatase inhibitors (Roche). For some experiments cells were fractionated as described previously [49]. Protein concentrations were determined and equalized, 5x SDS-PAGE sample buffer was added to each, and they were heated to 95°C for 10 minutes. Denatured proteins were resolved by SDS-PAGE and transferred to nitrocellulose membranes. The following antibodies were utilized: anti-BACH1 (FANCJ) (Sigma, B1310), anti-FANCD2 (Santa Cruz Biotechnology, sc-20022), anti-FANCC (Santa Cruz Biotechnology, sc-18110), anti-FANCI (Bethyl, A301–254A), anti-pChk1(S317) (Cell Signaling, #2344), anti-Beta-Actin (Santa Cruz Biotechnology, sc-47778), anti-Lamin B1 (Abcam, AB16048) and anti-GAPDH (Santa Cruz Biotechnology, sc-32233). Appropriate HRP-conjugated secondary antibodies (Sigma-Aldrich) were used to detect protein bands upon exposure to film (CL-Xposure, Thermo Scientific). Films were scanned and quantitated using Image-J [50].

### Immunoprecipitation

For immunoprecipitation, H1299 cells were treated as above and lysed with RIPA buffer (50 mM Tris-HCl pH 7.4, 150 mM NaCl, 1% Triton x-100, 1% Sodium deoxycholate, 0.1% SDS, 1 mM EDTA) plus protease and phosphatase inhibitors for whole cell protein extraction or CSK buffer and fractionated into chromatin and soluble protein extracts as above. For whole cell extraction, the cells were passed through a 21 gauge needle to insure complete lysis. Immunoprecipitation reactions were done using Protein A/G PLUS-Agarose IP Reagent (Santa Cruz Biotechnology) following the manufacturer's instructions.

### Cell cycle analysis and flow cytometry

Cells were grown and transfected with siRNAs as above and harvested at time points indicated. Cells were fixed with ice-cold 90% ethanol and cell cycle profiles were analyzed by flow cytometry, using a BD FACSCanto™ II (BD Biosciences) after propidium iodide (PI) staining as described previously [51].

### RNA extraction and qRT-PCR

Cells grown as above and treated as indicated were harvested for RNA extraction using Trizol (Ambion). Total RNA was quantitated and checked for purity, then converted into cDNA by the High Capacity RNA-to-cDNA Kit (Applied Biosystems) following manufacturer's instructions. Resultant cDNA was used for RT-PCR. The iTAQ™ Universal SYBR^®^ Green Supermix (BIO RAD) and FANCD2 and GAPDH PrimePCR primers purchased from BIO RAD (Cat# 10025036) were used for RT-PCR, according to supplied protocol. All samples were normalized to a GAPDH control.
